# A photochemical-responsive nanoparticle boosts doxorubicin uptake to suppress breast cancer cell proliferation by apoptosis

**DOI:** 10.1038/s41598-022-14518-x

**Published:** 2022-06-20

**Authors:** Ying Zhang, Kaiting Li, Xiaoyu Han, Qing Chen, Lan Shao, Dingqun Bai

**Affiliations:** 1 Department of Rehabilitation Medicine, The First Affiliated Hospital of Chongqing Medical University, Chongqing, 400016 China; 2 The Chongqing Key Laboratory of Translational Medicine in Major Metabolic Diseases, The First Affiliated Hospital of Chongqing Medical University, Chongqing, China

**Keywords:** Breast cancer, Nanoparticles, Photochemistry

## Abstract

In the course of chemotherapy for breast cancer, doxorubicin (DOX) is one of the most commonly prescribed agents. However, it has been recognized as clinically circumscribed on account of its poor selectivity and toxic reactions to normal tissues. Fortunately, the distinct merit of photochemical-responsive nanoparticle delivery systems to enhance cellular drugs uptake through localized concentration, adequate selective and minimizing systemic toxicity has aroused substantial interest recently. In this study, we synthesized photochemical-responsive nanoparticle by incorporating DOX, curcumin (CUR), and perfluorooctyl bromide (PFOB) into poly(lactic-co-glycolic acid) (PLGA) via double emulsification (DOX–CUR–PFOB–PLGA). The synthesized composite nanoparticles, which featured good ultrasound imaging, engendered photochemical activation for drug release when given laser irradiation. Cumulative release rates for DOX were 76.34%, and for CUR were 83.64%, respectively. Also, MCF-7 cells displayed significant intracellular DOX uptake and reactive oxygen species (ROS) levels, degraded cytoskeleton, and decreased cell growth and migration capacity. At the molecular level, cellular pAKT levels decreased, which resulted in downregulated HIF-1α and BAX/BCl-2 levels, leading to Caspase-3 activation and thus induction of apoptosis. Therefore, the photochemical-responsive nanoparticles possess the potential to elicit apoptosis in MCF-7 cells via enhanced DOX uptake.

## Introduction

Cancer is the leading cause of death worldwide, threatening human health. According to the global cancer statistics^1^, breast cancer has surpassed lung cancer as the predominant contributor to deaths in females in recent years. Among the current therapeutic modalities, doxorubicin (DOX), which is an antibiotic and effective antitumor agent, has been used widely in the treatment of breast cancer patients and has improved their prognosis. However, its low tissue selectivity has caused cardiotoxicity and other side-effects^2^, posing a severe threat to patients. Moreover, the presence of antibiotic resistance further restrains its application in patients^3^. As such, increasing the tumor selectivity of DOX, boosting its cellular uptake, and mitigating toxic side effects are pressing clinical issues.

Stimulus-responsive nanoparticle delivery systems can sense environmental changes or stimuli and respond accordingly to change the drug distribution in vivo in terms of space, time, and dose. These systems can lead to localized enrichment, retarded release, and increased stability of drugs upon appropriate stimulation, thus increasing drug effects and minimizing side effects^4,5^. Polymeric nanoparticles (NPs) are ideal carriers for drug delivery. Poly(lactic-co-glycolic acid) (PLGA) is one such polymeric NP that has been certified by the US Food and Drug Administration (FDA) due to its excellent biocompatibility and biodegradability. PLGA is non-toxic and suitable for sustained release and possesses favorable film and vesicle forming properties^6^. Regarding the numerous stimuli method, light^7^, as an external stimulus without temporal and spatial limitations, has attracted the most attention amidst controlled drug release systems due to its easy and remote adjustability while minimal damage to normal tissues. Inspired by photodynamic therapy (PDT) which uses cold laser irradiation of a photosensitizer to engender a series of photochemical reactions that trigger energy transfer from the photosensitizer to the surrounding oxygen to generate ROS^8,9^, we searched and tried to identify a suitable photosensitizer for localized cancer treatment using our NPs. However, most photosensitizers were found to be hydrophobic and prone to drug degradation. The third generation photosensitizers^10^, which can be loaded into either NPs or liposomes and have enhanced water solubility, can strengthen tumor targeting and improve photodynamic efficacy and have been proved to be good choices in many situations. Curcumin (CUR), a natural diphenolic compound extracted from the rhizome of Curcuma longa, stands out from the many photosensitizers due to its distinctive photosensitizing potential in photo- and acoustic-dynamic treatment against malignant tumors^11,12^ and its chemotherapeutic sensitizing effect^13^ at low dose. Hence, we chose CUR as the photosensitizer in our NPs. We prepared photochemical-responsive NPs by encapsulating DOX and CUR in PLGA, where the photosensitivity of CUR was employed to improve the PDT efficacy and its chemotherapeutic sensitizing effect was exploited to amplify the cytotoxicity of DOX.

Nevertheless, the hypoxic environment of the tumor site cannot accommodate enough oxygen demanded for photodynamic generation of singlet oxygen, resulting in a chemotherapeutic blind zone that constitutes a bottleneck for the optimal therapeutic effect of photochemical therapies. Thus, it is crucial to ameliorate the hypoxic environment and augment the local oxygen content within the tumors^14^. Perfluorooctyl bromide (PFOB), which is typically used as an ultrasound contrast agent^15^, is a member of fluorocarbons that has high oxygen affinity. The good biocompatibility and high oxygen adherence capacity of PFOB can significantly ameliorate internal tumor hypoxic conditions and increase the photodynamic efficacy^16,17^.

**Figure 1:**
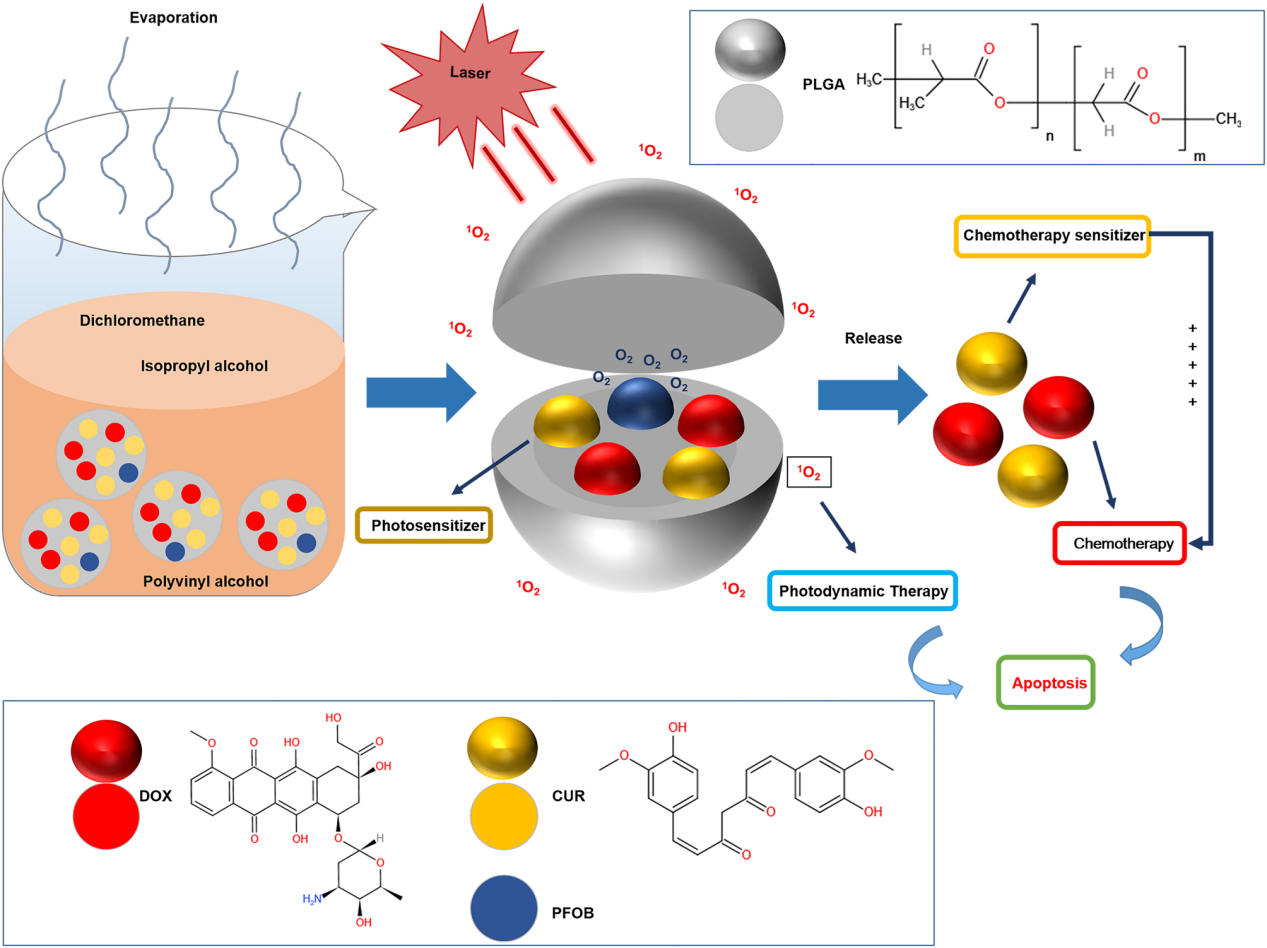
A schematic diagram of photochemical-responsive nanoparticles incorporating DOX, CUR, PFOB and PLGA and laser-triggered drug release induced apoptosis under the combined effect of PDT and chemotherapy. DOX: Doxorubicin; CUR: Curcumin; PLGA: Poly (lactic-co-glycolic acid); PFOB: perfluorooctyl bromide.

Therefore, we incorporated PFOB into PLGA NPs containing DOX and CUR to synthesize multifunctional photochemical-responsive NPs, which we named **DOX–CUR–PFOB–PLGA**, that significantly can increase intracellular DOX uptake and enable O_2_ self-sufficiency improve the efficacy of PDT (Fig. 1). In this study, we characterized different properties of the NPs, evaluated their in vitro drug release rate, and investigated their impact on breast cancer proliferation and the ultrasound imaging effect, which hopefully will lay a foundation for further investigations.

## Results

### Preparation process and characterization of nanoparticles

**Figure 2:**
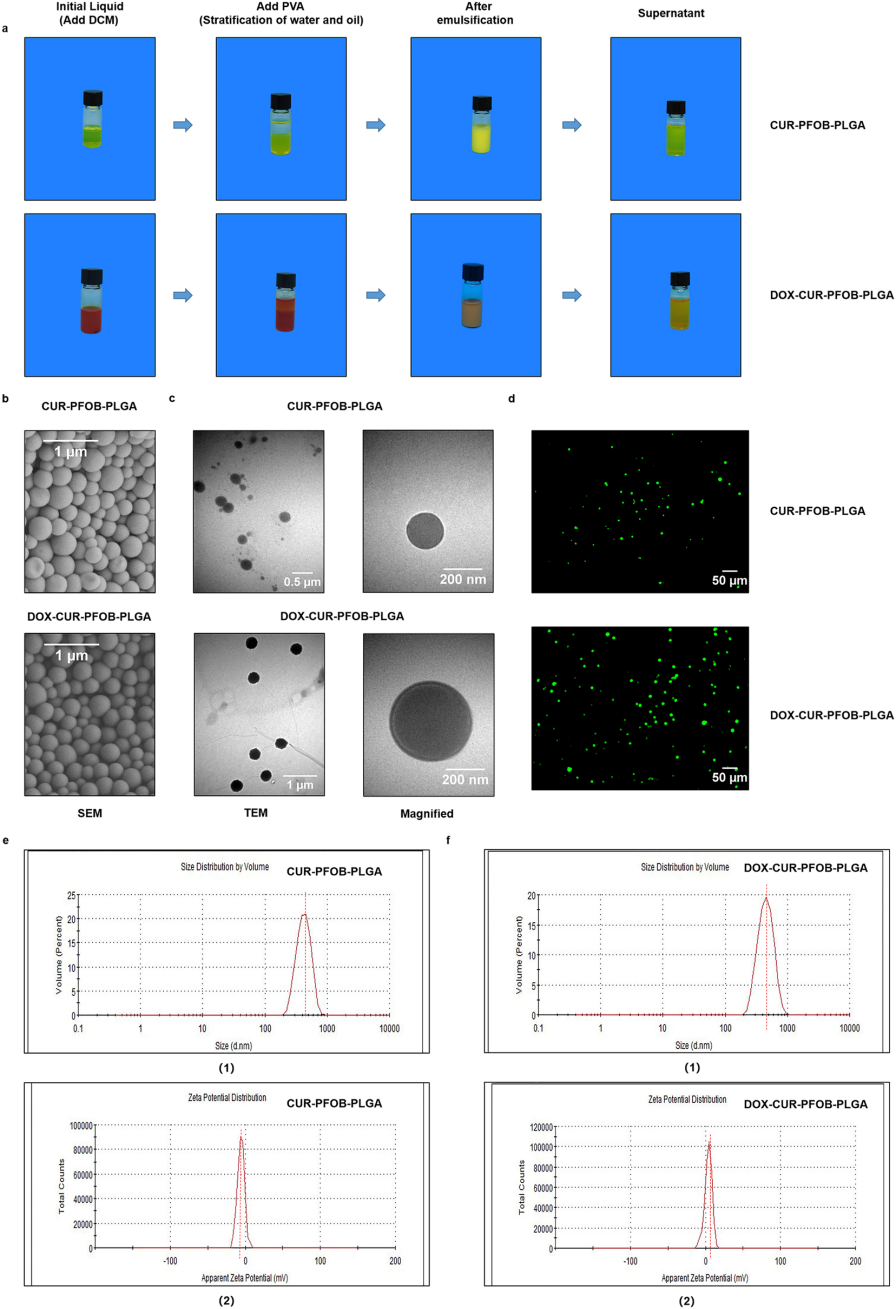
Preparation and morphological characterization of nanoparticles (NPs). (**a**) Digital camera photos of the preparation process of CUR–PFOB–PLGA and DOX–CUR–PFOB–PLGA NPs including dichloromethane (DCM) dissolution of the drugs, water–oil phase separation after polyvinyl alcohol (PVA) addition, emulsification, and the supernatant collected by centrifugation. (**b**) SEM images of CUR–PFOB–PLGA and DOX–CUR–PFOB–PLGA NPs; (**c**) TEM images of CUR–PFOB–PLGA and DOX–CUR–PFOB–PLGA NPs (The right images were the magnification of NPs.); (**d**) Morphology of NPs under inverted fluorescence microscope (The CUR in NPs showed green fluorescence by excitation.); (**e**) Size distribution (1) and Zeta potential (2) of CUR–PFOB–PLGA NPs by DLS (Mean diameter: 421.80 ± 111.50 nm); (**f**) Size distribution (1) and Zeta potential (2) of DOX–CUR–PFOB–PLGA NPs by DLS (Mean diameter: 409.77 ± 7.56 nm).

**Figure 3:**
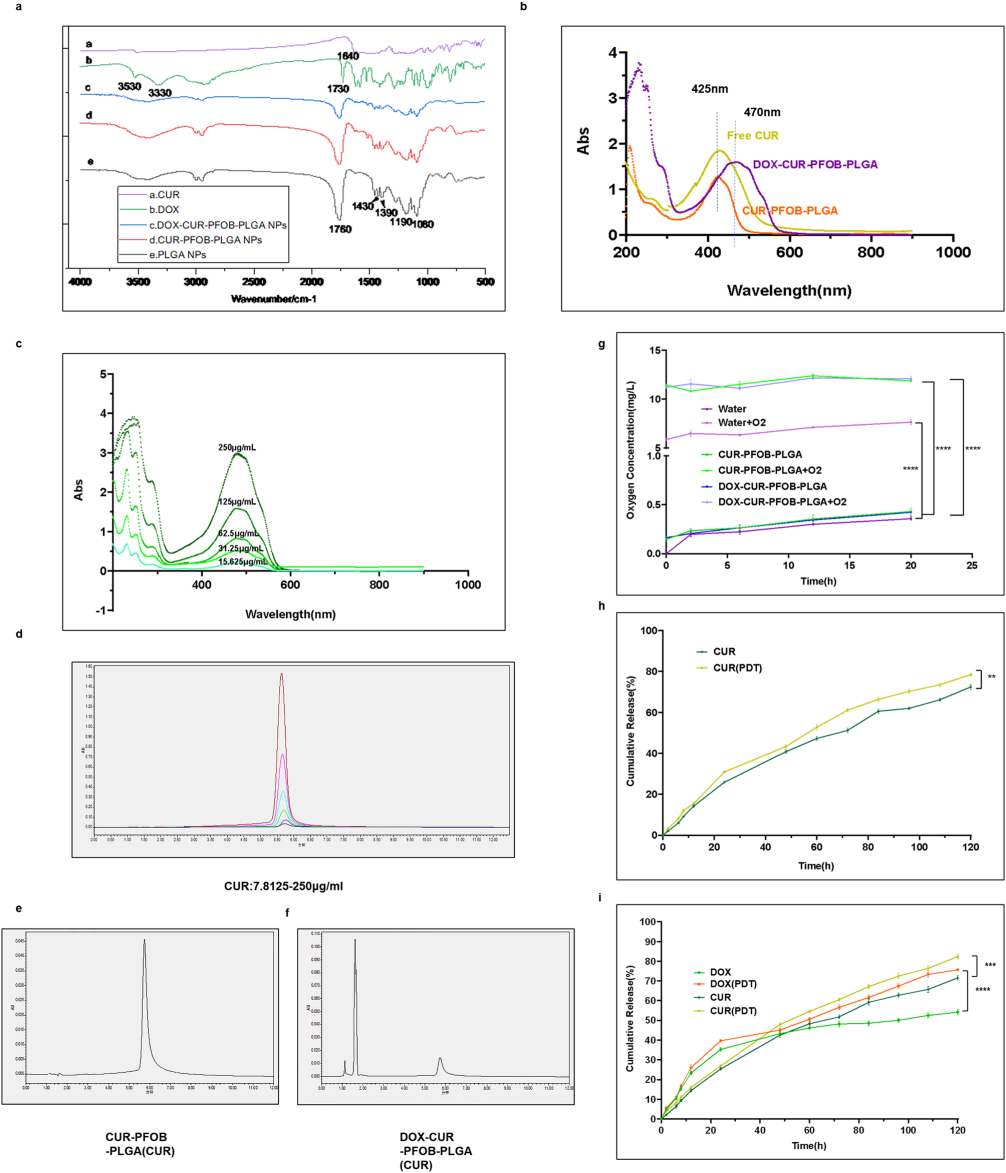
Characterization of nanoparticles. (**a**) Chemical structure analysis in FTIR (**a** CUR, **b** DOX, **c** DOX–CUR–PFOB–PLGA NPs, **d** CUR–PFOB–PLGA NPs, **e** PLGA NPs); (**b**) Absorbance of CUR, CUR–PFOB–PLGA NPs and DOX–CUR–PFOB–PLGA NPs by UV spectrophotometer (Horizontal coordinate: scanning wavelength, vertical coordinate: absorbance); (**c**) Absorbance of different concentrations of DOX under UV spectrophotometer (DOX: 15.625–250 μg/mL, horizontal coordinate is detection wavelength and vertical coordinate is absorbance); (**d**) Absorbance of different concentrations of CUR under HPLC (CUR: 7.8125–250 μg/mL, horizontal coordinate is absorption peak time, vertical coordinate is absorbance and the peak time of CUR is between 5 and 6 min); (**e**) Absorbance of CUR in CUR–PFOB–PLGA NPs at 5–6 min in HPLC; (**f**) Absorbance of CUR in DOX–CUR–PFOB–PLGA NPs at 5–6 min in HPLC; (**g**) Oxygen concentrations in NP solution with or without oxygen infusion at different time points were measured by portable dissolved oxygen meter (nanoparticles need to be oxygenated for 10 min); (**h**) Time dependent changes of the amount of CUR from CUR–PFOB–PLGA NPs by laser irradiation or not was measured by HPLC; (**i**) Cumulative release rates of DOX with or without laser irradiation from DOX–CUR–PFOB–PLGA NPs were measured by UV spectrophotometer and CUR were measured by HPLC. Statistics were analyzed by GraphPad Prism 8.0 (https://www.graphpad.com/updates); **P < 0.01; ***P < 0.001; ****P < 0.0001.

Two types of NPs, CUR–PFOB–PLGA and DOX–CUR–PFOB–PLGA, were prepared by incorporating CUR and PFOB, or DOX, CUR and PFOB into PLGA, respectively, using the water-in-oil-in-water (W1/O/W2) double-emulsification/solvent evaporation method as described by Kim et al.^18^. The preparation process of both NPs were photographed by digital camera (Fig. 2a). The obtained NPs were observed using transmission electron microscopy (TEM) and scanning electron microscopy (SEM). According to SEM images, the CUR–PFOB–PLGA and DOX–CUR–PFOB–PLGA NPs both showed spherical shapes and uniform sizes (Fig. 2b), which were 254 ± 4.44 (mean ± standard deviation (SD)) nm and 333 ± 4.04 nm respectively under transmission electron microscopy (TEM) observation (Fig. 2c). These results suggested that the slightly increased particle size of DOX–CUR–PFOB–PLGA NPs may be attributed to the addition of DOX. Under the inverted fluorescence microscope, the existence of CUR in NPs were detected by the immunofluorescence reaction and the green fluorescent NPs were exhibited as regular circular shapes (Fig. 2d), consistent with the TEM and SEM results. The NPs sizes of CUR–PFOB–PLGA (Fig. 2e-(1)) and DOX–CUR–PFOB–PLGA (Fig. 2f-(1)) NPs measured by the dynamic light scattering (DLS) method were 421.80 ± 111.50 nm and 409.77 ± 7.56 nm, which were slightly larger than those measured under the SEM and TEM because the hydrodynamic diameter of the NPs in solution. The Zeta potentials of the CUR–PFOB–PLGA and DOX–CUR–PFOB–PLGA NPs were −5.80 ± 0.32 mV (Fig. 2e-(2)) and 4.00 ± 0.13 mV (Fig. 2f-(2)). The difference in the Zeta potentials was probably due to the presence of positively charged amino groups (Fig. 1) in DOX. In the Fourier transform infrared spectrometer (FTIR) (Fig. 3a), the characteristic peaks of DOX at 1730 cm^−1^, 3330 cm^−1^ and 3530 cm^−1^ are related to the stretching vibration of carbonyl (**C=O**), hydroxyl (**–OH**), and amino (**–NH**_**2**_); CUR at 1640 cm^−1^ is an important sign of the benzene ring backbone (**C=C**) stretching; while the PLGA NPs at 1760 cm^−1^ represents the stretching vibration of **C=O** in the ester bond, 1430 cm^−1^ and 1390 cm^−1^ correspond to the swinging vibration of the saturated **C–H** bond, 1190 cm^−1^ and 1080 cm^−1^ belong to the **C–O** stretching vibration of the ester group, and near 3000 cm^−1^, the stretching vibration of methyl (**–CH**_**3**_) and methylene (**–CH**_**2**_) appear^19^. The absence of new characteristic peaks in nanoparticles c and d, just a simple superposition of the respective absorption peaks, suggested that the compounds may be physically mixed with each other. Besides, ultraviolet (UV) spectrophotometer demonstrated strong absorption peaks at 425 nm for both free CUR solution and the supernatant of CUR–PFOB–PLGA NPs after centrifugation and the absorption peak was inhibited in the supernatant (Fig. 3b). Similarly, UV spectrophotometer displayed strong peaks at 480 nm for free DOX solution (Fig. 3c) and at 470 nm for the supernatant of DOX–CUR–PFOB–PLG**A** NPs (Fig. 3b). The difference (i.e., 480 nm vs. 470 nm) in DOX absorption peaks may be due to the interference of CUR. These results all suggested the successful synthesis of such composite nanoparticles. Later, in order to exclude the interaction effect of the absorption peaks of DOX and CUR (i.e., 480 nm vs. 425 nm), we plotted the standard curve by the absorbance of different concentrations of DOX and CUR with UV spectrophotometer (Fig. 3c) and high-performance liquid chromatography (HPLC) (Fig. 3d) respectively to indirectly calculate their loading efficiency (LE) and encapsulation efficiency (EE) in NPs. The LE and EE of CUR were (3.36 ± 0.33)% and (82.80 ± 2.51)%, respectively, in the CUR–PFOB–PLGA NPs (Fig. 3e), and were (6.65 ± 0.01)% and (79.78 ± 0.15)%, respectively, in the DOX–CUR–PFOB–PLGA NPs (Fig. 3f). Based on Fig. 3b and c, the LE and EE of DOX were (5.90 ± 0.04)% and (70.80 ± 0.47)%, respectively, in the DOX–CUR–PFOB–PLGA NPs. Also, a portable dissolved oxygen meter was used to measure the ability of NPs to adhere to oxygen. Two types of NPs were dissolved in the ultrapure water. As shown in Fig. 3g, the oxygen concentrations in the ultrapure water, CUR–PFOB–PLGA and DOX–CUR–PFOB–PLGA NPs were within 0.5 mg/L. But in the ultrapure water with oxygen infusion increased by 5.87 ± 0.15 mg/L at the beginning, and gradually increased to 7.64 ± 0.22 mg/L within 20 h (h) (*P* < 0.001). In comparison, the oxygen levels in the CUR–PFOB–PLGA and DOX–CUR–PFOB–PLGA NPs with oxygen infusion increased by 11.30 ± 0.11 mg/L and 11.05 ± 0.11 mg/L, respectively, at the beginning, and this value was maintained at around 11 mg/L in the subsequent 20 h (*P* < 0.0001), indicating that the two types of NPs have high oxygen adherence capacity and low oxygen release rate in vitro.

### Drug release in vitro

The release behavior of DOX and CUR from the NPs was measured using the dialysis method^20^ and each release data point were represented by the cumulative percentage of drugs released over an interval of time. As shown in Fig. 3h, the cumulative release of CUR was 72.40 ± 1.22%. It was released rapidly in the first 24 h, slowly in the next 24 h, and entered a long-acting slow-release phase at 48–120 h. The release pattern for CUR was the same when the NPs were given laser irradiation at a wavelength of 425 nm and an intensity of 40 mW/cm^2^ for 150 s with a cumulative release rate of 78.47% (*P* < 0.01). In the DOX–CUR–PFOB–PLGA NPs, the total release of DOX increased from 55.76 to 76.34% (*P* < 0.0001) and the total release rate of CUR increased from 71.63 to 82.36% (*P* < 0.001) before and after laser irradiation (Fig. 3i). The increase in drug release upon photochemical activation (i.e., laser irradiation) may be due to the energy transfer caused by laser irradiation of the photosensitizer^21^. In order to scientifically analyze the in vitro release behavior of DOX and CUR, relevant kinetic fits^22^ (Zero-order kinetic, First-order kinetic, Higuchi and Ritger-peppas) were performed to evaluate the drug release mechanism. Based on the fitting results (Table 1). When R^2^ is close to 1, the best fit is achieved. The release profiles of CUR in both NPs with or without laser irradiation were dominated by First-order kinetic (R^2^≈1), demonstrating that the release pattern of CUR belongs to a slow release process and the next best fit was the Ritger-peppas model. For spherical nanoparticles, the release mechanism of nanoparticles can be judged according to the release index n in the Ritger-peppas model. The n of CUR is 0.6–0.7 (0.43 < *n* < 0.85), indicating that CUR release is a synergistic effect of Fickian diffusion and erosion mechanisms. Compared with CUR, the DOX release from DOX–CUR–PFOB–PLGA NPs without laser irradiation showed the similar results (R^2^ = 0.993), but the n of DOX was 0.4222 (n < 0.43) which explained that the release behavior of DOX was Fickian diffusion. Meanwhile, after laser irradiation, the best release behavior of DOX transferred from First-order kinetic to Higuchi model may be due to diffusion release controlled by the carrier^23^, while the n was 0.5306 (*n* > 0.43) to form a mechanism of synergistic action of Fickian diffusion and erosion. Therefore, such composite nanoparticles can not only achieve a slow release efficacy especially when given laser irradiation, but also increase the maximum drug release with the combination of laser, providing a novel concept for photochemical-responsive nanoparticle systems.

**Table 1:** Mathematical fitting results of NPs.

	Drugs	Mathematical model	Fitting equation	R^2^
CUR–PFOB–PLGA	CUR	Zero-order kinetic	M_t_ = 0.6034t + 5.5035	0.9680
		First-order kinetic	M_t_ = 89.6462(1 − e^−0.0128t^)	**0.9967**
		Higuchi	M_t_ = 7.1287t^1/2^ − 7.8506	0.9860
		Ritger-peppas	M_t_ = 2.6778t^0.6917^	**0.9939**
	CUR–PDT	Zero-order kinetic	M_t_ = 0.6600t + 7.1648	0.9630
		First-order kinetic	M_t_ = 94.1496(1 − e^−0.0143t^)	**0.9965**
		Higuchi	M_t_ = 7.8229t^1/2^ − 7.5949	0.9878
		Ritger-peppas	M_t_ = 3.4059t^0.6622^	**0.9936**
DOX–CUR–PFOB–PLGA	DOX	Zero-order kinetic	M_t_ = 0.4141t + 12.8657	0.8202
		First-order kinetic	M_t_ = 51.3105(1 − e^−0.0445t^)	**0.9925**
		Higuchi	M_t_ = 5.1895t^1/2^ + 1.9057	0.9516
		Ritger-peppas	M_t_ = 7.5834t^0.4222^	0.9615
	DOX–PDT	Zero-order kinetic	M_t_ = 0.5873t + 11.7851	0.9288
		First-order kinetic	M_t_ = 74.7575(1 − e^−0.0238t^)	0.9642
		Higuchi	M_t_ = 7.0706t^1/2^ − 2.0089	**0.9858**
		Ritger-peppas	M_t_ = 5.9780t^0.5306^	**0.9854**
	CUR	Zero-order kinetic	M_t_ = 0.5997t + 5.8184	0.9637
		First-order kinetic	M_t_ = 85.9768(1 − e^−0.0138t^)	**0.9980**
		Higuchi	M_t_ = 7.1036t^1/2^ − 7.5670	0.9873
		Ritger-peppas	M_t_ = 2.8498t^0.6778^	**0.9933**
	CUR–PDT	Zero-order kinetic	M_t_ = 0.6897t + 6.6128	0.9716
		First-order kinetic	M_t_ = 103.2859(1 − e^−0.0127t^)	**0.9989**
		Higuchi	M_t_ = 8.1374t^1/2^ − 8.5836	0.9865
		Ritger-peppas	M_t_ = 3.1023t^0.6898^	**0.9960**

The fitted equations were all plotted by the Origin 2021 software (https://www.originlab.com/2021).

Significant values are in [bold].

### Biocompatibility of nanoparticles

#### CCK-8

**Figure 4:**
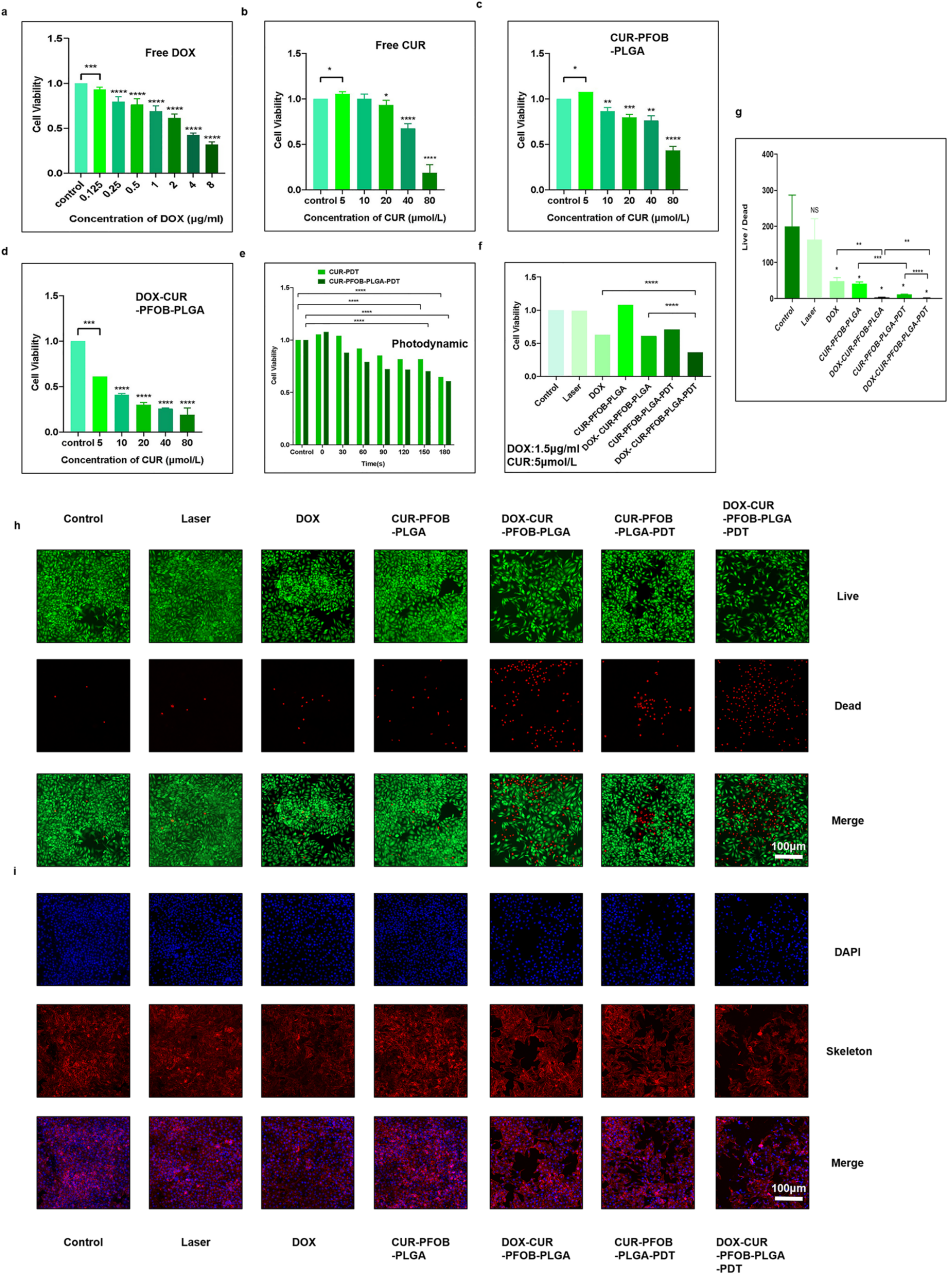
MCF-7 cell viability. (**a**) Effect of 0.125–8 μg /mL DOX on MCF-7 cell viability; (**b**) Effect of 5–80 μmol/L CUR on MCF-7 cell viability; (**c**) Effect of 5–80 μmol/L CUR–PFOB–PLGA NPs on MCF-7 cell viability; (**d**) Effect of 5–80 μmol/L DOX–CUR–PFOB–PLGA NPs on MCF-7 cell viability; (**e**) Effect of 0–150 s laser irradiation in combination with 5 μmol/L CUR or CUR–PFOB–PLGA NPs on MCF-7 cell viability; (**f**) Effect of 1.5 μg/mL DOX, 5 μmol/L CUR, CUR–PFOB–PLGA NPs and DOX–CUR–PFOB–PLGA NPs and 150 s laser irradiation alone or in combination on the viability of MCF-7 cells; (**g**) The ratio of the counts of live cells to dead cells in MCF-7 cells; (**h**) Live/Dead analysis of MCF-7 cells under LSCM. (Live cells were stained green while dead cells appeared red.); (**i**) The LSCM images of MCF-7 cells (The cytoskeleton was stained in red and the nucleus was blue.). (NS, no significant difference; *P < 0.05; **P < 0.01; ***P < 0.001; ****P < 0.0001).

The NPs were saturatedly loaded with oxygen by oxygen infusion for 10 min before use. In the CCK-8 assay, we found that the cell viability in free DOX treated cells was negatively concentrated with DOX concentration (Fig. 4a) (*P* < 0.0001). When the CUR concentration was between 5 and 20 μmol/L, the cell viability in free CUR (Fig. 4b) and the CUR–PFOB–PLGA NPs (Fig. 4c) treated groups was higher than 80%. When the CUR concentration was higher than 20 μmol/L, the cell viability in both groups decreased gradually. However, when CUR was at the same concentration, the DOX–CUR–PFOB–PLGA NPs presented stronger cytotoxicity (Fig. 4d). To avoid the cardiotoxicity and drug resistance effects caused by prolonged utilization of DOX^24^ and considering that CUR was used as a sensitizer, we used relatively low CUR concentrations in all groups for subsequent experiments. At this low CUR concentration (5 μmol/L), no lethal effects were observed in cells in both the free CUR and the CUR–PFOB–PLGA groups. As shown in Fig. 4e, the cell survival rate declined with increasing laser irradiation time. For example, at an irradiation time between 0 and 150 s, the survival rate was 80% and at an irradiation time between 150 and 180 s, the survival rate was lower than 65%. We used an irradiation time of 150 s with DOX–CUR–PFOB–PLGA NPs to investigate the cell viability. As shown in Fig. 4f, laser irradiation alone did not affect the cell survivability at all. The cell survival rate was lowest in the DOX–CUR–PFOB–PLGA–PDT group, followed by the CUR–PFOB–PLGA–PDT group and DOX–CUR–PFOB–PLGA and the free DOX groups.

#### Photochemical-responsive DOX–CUR–PFOB–PLGA NPs decrease the live/dead cell ratio

The principle of live/dead staining is that esterase activity and plasma membrane integrity are indicated by green fluorescent calcein-AM staining and red fluorescent ethidium homodimer-1 staining, respectively, to indicate live and dead cells. From the laser scanning confocal microscope (LSCM) images (Fig. 4h), we analyzed the number of live and dead cells by Image J and used Prism to analyze the data for live/dead cell ratio (Fig. 4g). There was no significant difference between the laser-irradiated and non-laser-irradiated (control) groups. The live/dead cell ratio in the free DOX group was decreased and in the DOX–CUR–PFOB–PLGA group was significantly decreased (*P* < 0.01), indicating a good tumor growth inhibitory effect of the DOX–CUR–PFOB–PLGA NPs. Furthermore, the ratio were lower than those in the same groups without laser irradiation in the CUR–PFOB–PLGA–PDT (*P* < 0.01) and DOX–CUR–PFOB–PLGA–PDT (*P* < 0.001) groups. When comparing the CUR–PFOB–PLGA–PDT and DOX–CUR–PFOB–PLGA–PDT groups, the DOX–CUR–PFOB–PLGA–PDT group exhibited an reduced live/dead cell ratio (*P* < 0.0001). All these results indicate that the photochemical-responsive NPs combined with laser irradiation possess superior ability to inhibit the growth of MCF-7 cells.

#### Photochemical-responsive DOX–CUR–PFOB–PLGA NPs disrupt the skeleton structure

The directional migration of cells is a fundamental characteristic of biological processes. In this process, the lamellar pseudopods at the cell fronts form drawn cytosomes. Polymerization, depolymerization and reassembly of the cytoskeleton and related binding proteins form the material basis of this process^25,26^. As shown in Fig. 4i, actins in the control group, laser irradiation group and CUR–PFOB–PLGA group underwent polymerization, Compared with cells in the CUR–PFOB–PLGA group, cells in the CUR–PFOB–PLGA–PDT group showed slight depolymerization as indicated by reduced dotted fluorescence signals. The polymerization ability of actin was inhibited in the free DOX and DOX–CUR–PFOB–PLGA groups. Surprisingly, after laser irradiation, the cytoskeleton had curtailed polymerization ability and almost completely disappeared in cells in the DOX–CUR–PFOB–PLGA–PDT group indicated by the disrupted filamentous structure. The skeletal system in cells is the support structure for living cells. So, this photochemical-responsive DOX–CUR–PFOB–PLGA has been proven to weaken the cellular activity of MCF-7 cells by more effectively depolymerizing the cytoskeleton.

### Photochemical-responsive DOX–CUR–PFOB–PLGA NPs boost DOX uptake by MCF-7 cells

**Figure 5:**
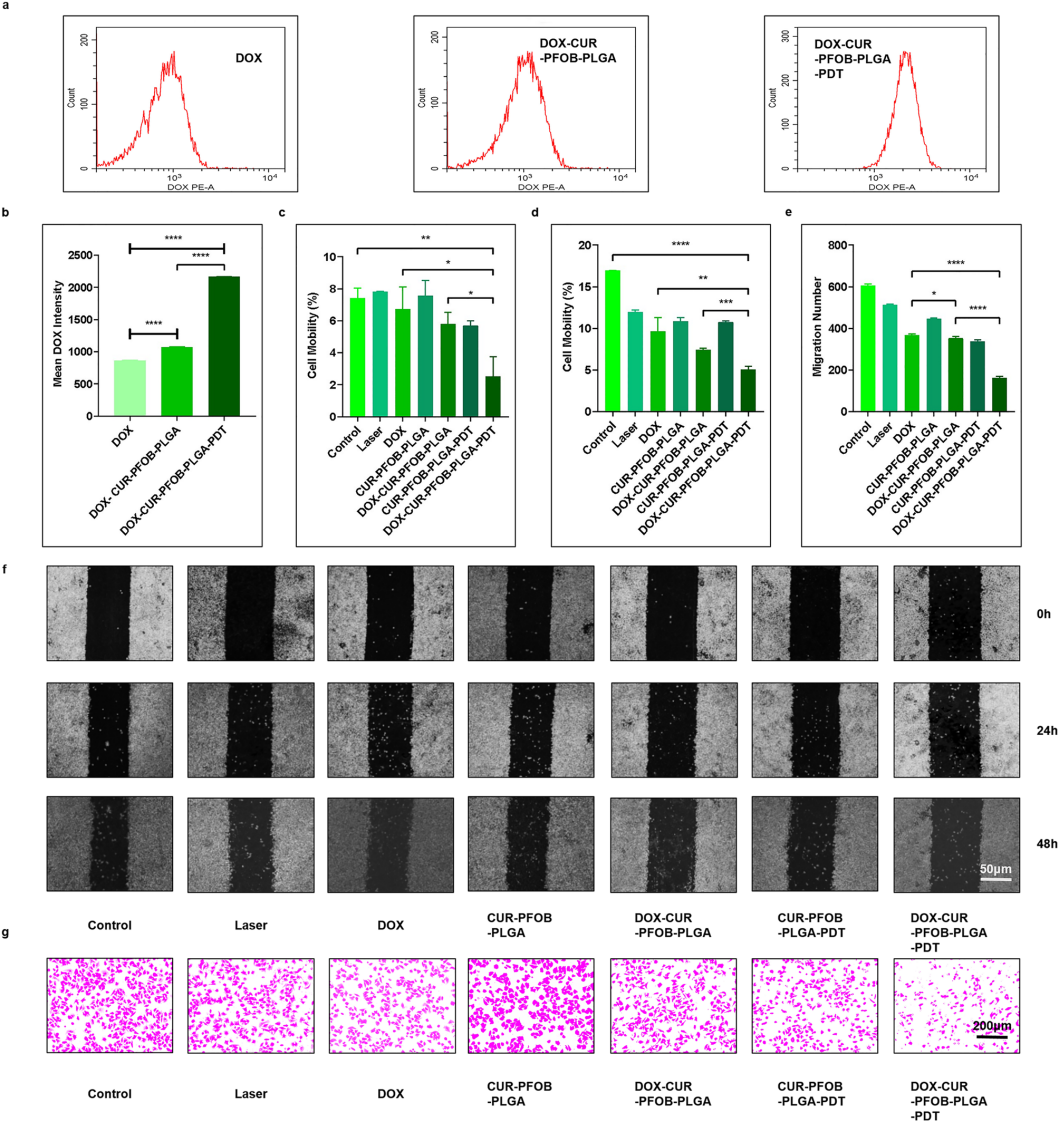
Examination of intracellular uptake and migration characteristics of MCF-7 cells. (**a**) FCM detection of intracellular DOX fluorescence intensity; (**b**) Statistics of DOX fluorescence intensity; (**c**) Statistics of MCF-7 cell mobility at 24 h; (**d**) Statistics of MCF-7 cell mobility at 48 h; (**e**) Statistics of MCF-7 cell migration numbers; (**f**) Images of MCF-7 wound scratches under inverted fluorescence microscope at 0 h, 24 h and 48 h; (**g**) Images of crystal violet staining of migrating cells under inverted fluorescence microscope. All statistics above were analyzed by Image J 1.46 r (https://imagej.nih.gov/ij/docs/guide/146.html) and GraphPad Prism 8.0 (https://www.graphpad.com/updates), *P < 0.05; **P < 0.01; ***P < 0.001; ****P < 0.0001).

To explore the reason why DOX–CUR–PFOB–PLGA NPs combined with laser irradiation inhibit the growth of MCF-7 cells, we examined the capacity of MCF-7 cells to uptake DOX. When the same concentrations of DOX were applied to MCF-7 cells, the fluorescence intensity of DOX, which indicates cellular DOX uptake, was highest in the DOX–CUR–PFOB–PLGA–PDT group, followed by the DOX–CUR–PFOB–PLGA group and the free DOX group (Fig. 5a) under flow cytometry (FCM). Thus, we proved that the NPs could facilitate the DOX cellular uptake, and the laser irradiation treatment could make the effects even stronger (*P* < 0.0001). The quantitative analysis of intracellular fluorescence intensity of DOX is shown in Fig. 5b.

### Photochemical-responsive DOX–CUR–PFOB–PLGA NPs inhibit the migration of MCF-7 cells

#### Wound healing assay

In the wound healing assay, the cells could perceive the “scarred wound” without any external chemokine interference and move toward the “wound” area^27^. Figure 5f depicts the “wound healing” status of cells in each group at 0 h, 24 h and 48 h. At 24 h and 48 h, cells in the control group, laser irradiation group, and CUR–PFOB–PLGA group gradually migrated from the edge of the scratch to the middle. Compared to cells in the free DOX and DOX–CUR–PFOB–PLGA groups, they filled the “wound” area gradually at 24 h. Interestingly, the scratched gap in the free DOX group showed a pronounced diminution while the gap in the DOX–CUR–PFOB–PLGA group displayed only a slight diminution at 48 h, indicating that the inhibition effect on cell migration was substantially weakened over time in the free DOX group and the DOX–CUR–PFOB–PLGA NPs can reverse this effect to some extent. In comparison, the cell migration rates in the CUR–PFOB–PLGA–PDT group was even lower than that in the CUR–PFOB–PLGA group at 24 h (Fig. 5c), however, the migration rates in both groups were similar at 48 h (Fig. 5d). In contrast, the gap in the DOX–CUR–PFOB–PLGA–PDT group was almost not changed at 24 h compared with that in the free DOX and DOX–CUR–PFOB–PLGA groups; even at 48 h, the mobility was only (5.07 ± 0.3)%, indicating an extremely weak migration capacity for cells in the DOX–CUR–PFOB–PLGA–PDT group. These results suggest that such photochemical-responsive DOX–CUR–PFOB–PLGA NPs combined with PDT provide a considerable advantage over free DOX or isolated applications in curbing cell migration.

### Transwell assay

With quantitative analysis of migration numbers (Fig. 5e) after filming at 200 × magnification under the inverted fluorescence microscope (Fig. 5g), we identified 605 migrated cells in the control group, 512 migrated cells in the laser irradiation group, 368 migrated cells in the free DOX group, 447 migrated cells in the CUR–PFOB–PLGA group, 353 cells in the DOX–CUR–PFOB–PLGA group, 338 migrated cells in the CUR–PFOB–PLGA–PDT group and 163 migrated cells in the DOX–CUR–PFOB–PLGA–PDT group. Indeed, when laser irradiation was applied to the DOX–Cur–PFOB–PLGA NPs, the migration ability of MCF-7 cells was significantly inhibited (*P* < 0.0001). In summary, the DOX–CUR–PFOB–PLGA NPs are promising photosensitizers for restraining breast cancer cell migration.

### Photochemical-responsive DOX–CUR–PFOB–PLGA NPs reduce cell proliferation capacity

**Figure 6:**
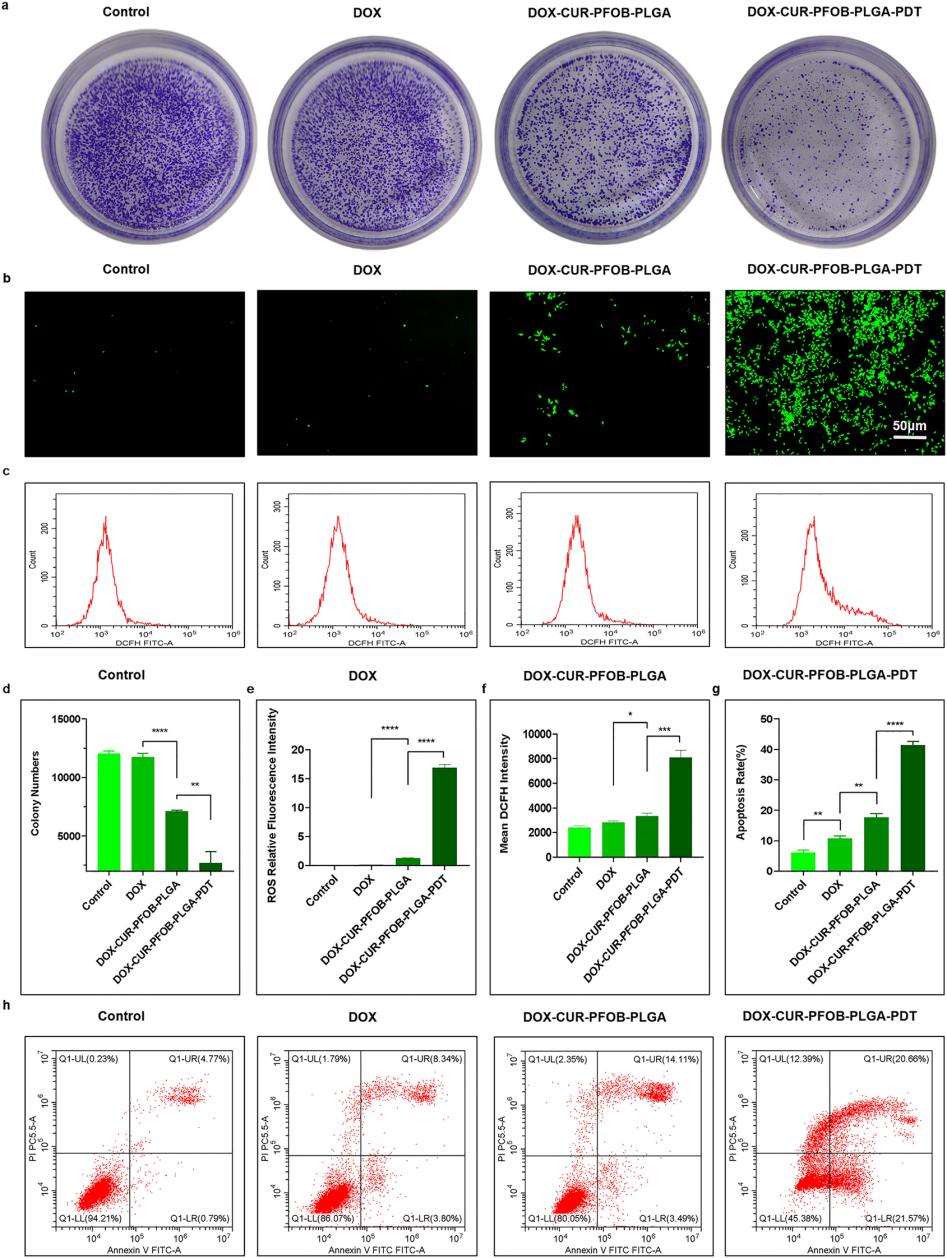
Proliferation、ROS generation capacity and apoptosis of MCF-7 cells on DOX–CUR–PFOB–PLGA NPs combined with PDT. (**a**) Digital camera photos of the survival and proliferation behavior of MCF-7 cells cultured with DOX, DOX–CUR–PFOB–PLGA or DOX–CUR–PFOB–PLGA and PDT for 14 days (The cells were stained with crystal violet); (**b**) Images of the generation of ROS by the inverted fluorescence microscope; (**c**) FCM detection of intracellular DCFH fluorescence intensity; (**d**) Statistics of colony numbers of MCF-7 cells; (**e**) Statistics of ROS relative fluorescence intensity; (**f**) Statistics of DCFH fluorescence intensity; (**g**) Statistics of the total apoptosis rate in early and late stages. (**h**) Apoptosis incidence quantified by flow cytometry through Annexin V and PI staining (Upper left quadrant (UL): cellular debris, lower left quadrant (LL): live cells, upper right quadrant (UR): late apoptotic cells, lower right quadrant (LR): early apoptotic cells.). Data are expressed as mean ± standard deviation (SD) and analyzed by the GraphPad Prism 8.0 (https://www.graphpad.com/updates) and Image J 1.46 r (https://imagej.nih.gov/ij/docs/guide/146.html) software, *P < 0.05; **P < 0.01; ***P < 0.001; ****P < 0.0001.

Subsequently, we specifically examined the effect of DOX–CUR–PFOB–PLGA NPs with laser irradiation on the proliferation of MCF-7 cells. After a 14-day free growth (Fig. 6a), cells in the control group showed an insane clone-forming competence, with a ~ 15-fold increase in the number of clones, reaching a final number of 12,043. In contrast, cells in the free DOX group exhibited slightly fewer clones, which could be due to that DOX could initially inhibit tumor growth but inhibition became weaker and weaker over time due to the gradually gained cellular resistance capacity^24^. These results were consistent with those in the scratch test. In the DOX–CUR–PFOB–PLGA–PDT group, the colony number was only ~ 2698, indicating a strong proliferation inhibition compared to the DOX–CUR–PFOB–PLGA group (*P* < 0.01) (Fig. 6d). This inhibition may be explained that possibly laser stimulation promotes the release of DOX from DOX–CUR–PFOB–PLGA NPs and accelerates efficient uptake of DOX by MCF-7 cells, thus leading to inhibition of colony formation.

### Photochemical-responsive DOX–CUR–PFOB–PLGA NPs enhance ROS generation capacity

ROS is a by-product of oxygen metabolism and plays an essential role as a second messenger in cell signaling under normal situations. Tumor cells are more sensitive to the accumulation of ROS, which can cause apoptosis^28^. ROS levels can be detected by the 2’–7’dichlorofluorescin diacetate (DCFH-DA) probe, as indicated by the fluorescence intensity of dichlorofluorescein (DCF). As shown in Fig. 6b, the ROS levels were low in the control group and free DOX group, and slightly higher in the DOX–CUR–PFOB–PLGA group, which may be due to that there was sufficient oxygen supply in the latter. In comparison, the ROS levels were much higher in the DOX–CUR–PFOB–PLGA–PDT group, and the quantitative analysis of fluorescence also indicated a similar trend (Fig. 6e). As shown in Fig. 6c, these conclusions were further confirmed by the flow cytometric analyses. In more detail (Fig. 6f), the analyses showed that ROS levels were low in the free DOX group, a little stronger in the DOX–CUR–PFOB–PLGA group (*P* < 0.05), and much stronger in the DOX–CUR–PFOB–PLGA–PDT group (*P* < 0.001).

### Photochemical-responsive DOX–CUR–PFOB–PLGA NPs raise apoptosis rate

As shown in Fig. 6h, the apoptosis ratios analyzed by Prism software (Fig. 6g) were (6.11 ± 0.69)% for the control group, (10.72 ± 0.73)% for the free DOX group, (17.31 ± 0.22)% for the DOX–CUR–PFOB–PLGA group, and (42.17 ± 0.90)% for the DOX–CUR–PFOB–PLGA–PDT group. We can see that the apoptosis ratio increased only ~ 4% in the free DOX group, while increased ~ 11% in the DOX–CUR–PFOB–PLGA group, which was significant (*P* < 0.05) when compared with the control group. The apoptosis ratio increased hugely (~ 36%) in the DOX–CUR–PFOB–PLGA group with irradiation treatment (*P* < 0.0001), indicating that laser irradiation on the DOX–CUR–PFOB–PLGA NPs could promote DOX uptake and thus result in much stronger apoptosis.

### Western blot

**Figure 7:**
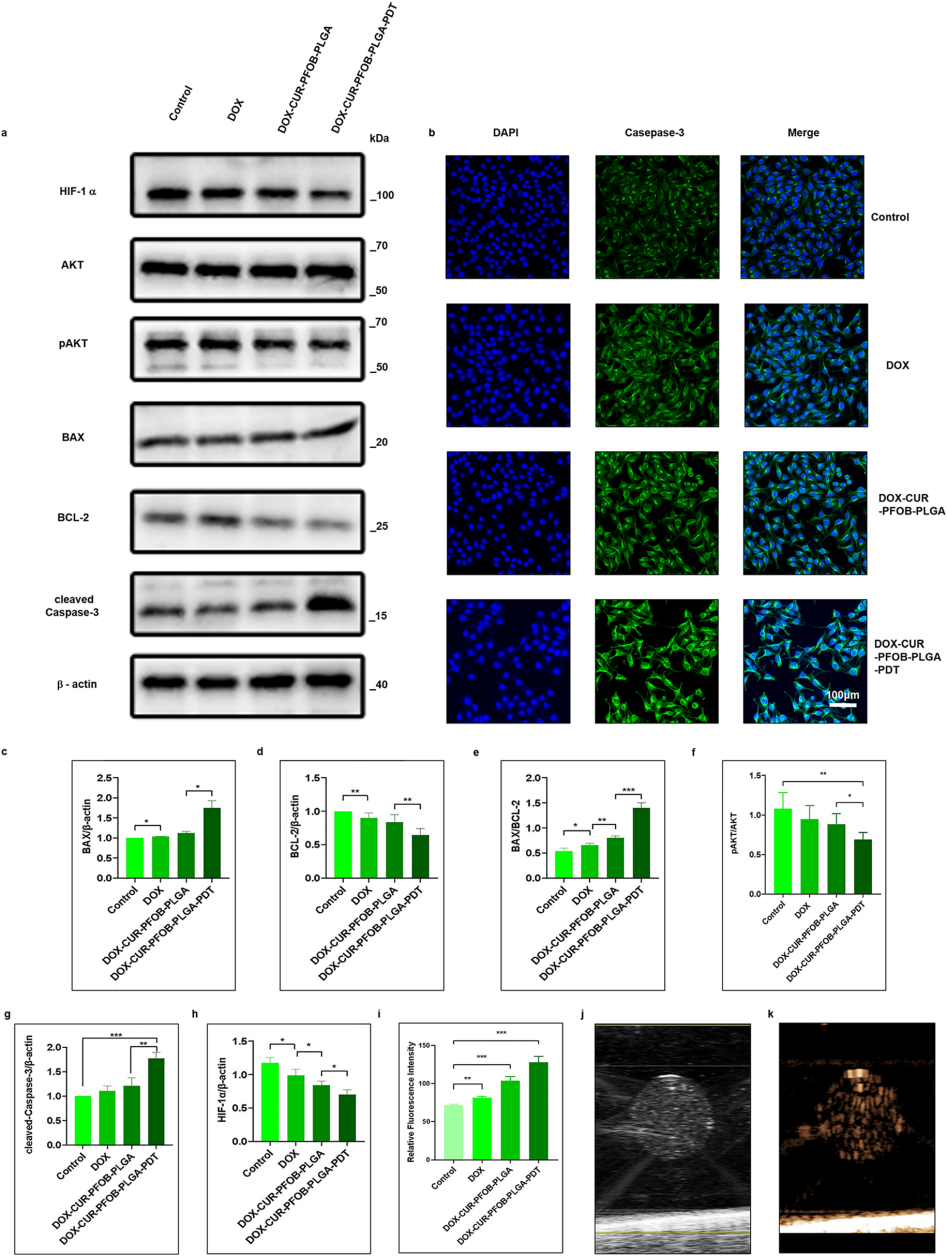
Molecular biology detection of MCF-7 cells and ultrasound imaging of DOX–CUR–PFOB–PLGA NPs by HIFU. (**a**) Western blot detection for HIF-1α, AKT, p-AKT, BAX, BCL-2, cleaved Caspase-3 and β-actin protein expressions, The full-length gels of all cropped blots combined are shown in Supplementary Figure S1; (**b**) The effects of DOX–CUR–PFOB–PLGA NPs on the expression of Caspase-3 (green) in MCF-7 cells incorporated with DOX, DOX–CUR–PFOB–PLGA or DOX–CUR–PFOB–PLGA and PDT, The nucleus was counterstained with DAPI (blue). (c) Quantitative analysis of the expression of BAX/β-actin; (**d**) Quantitative analysis of the expression of BCL-2/β-actin; (e) Quantitative analysis of the expression of BAX/BCL-2; (**f**) Quantitative analysis of the expression of pAKT/AKT; (**g**) Quantitative analysis of the expression of cleaved Caspase-3/β-actin; (**h**) Quantitative analysis of the expression of HIF-1α/β-actin. (**i**) Quantitative analysis of the relative fluorescence intensity of Caspase-3; (**j**) The B-mode image of DOX–CUR–PFOB–PLGA NPs by ultrasonic diagnostic apparatus; (**k**) The imaging mode image of DOX–CUR–PFOB–PLGA NPs by ultrasonic diagnostic apparatus. The expression of the proteins was quantified by Image J 1.46 r (https://imagej.nih.gov/ij/docs/guide/146.html) and Graphpad Prism 8.0 (https://www.graphpad.com/updates), *P < 0.05; **P < 0.01; ***P < 0.001.

As shown in Fig. 7a, the relative expression levels of total AKT were not different among the groups, while the pAKT protein levels were highest in the control group, and lowest in DOX–CUR–PFOB–PLGA–PDT group. The ratio of pAKT to AKT progressively declined (Fig. 7f). Also, the expression level of BAX (Fig. 7c) gradually increased while BCL-2 (Fig. 7d) gradually decreased (i.e., BAX/BCL-2 upregulation (Fig. 7e)). In addition, HIF-1α (Fig. 7h) was down-regulated and cleaved Caspase-3 increased (Fig. 7g) in the DOX–CUR–PFOB–PLGA–PDT group, further confirming that laser irradiation on the NPs could promote the apoptosis significantly.

### Immunofluorescence

We also examined the expression of Caspase-3 in MCF-7 cells by immunofluorescence staining. The results were consistent with those indicated by Western blot. Caspase-3 is an apoptotic protein located in the cytoplasm. As shown in Fig. 7b, the fluorescence intensity in the control group was weakest, followed by the DOX group and DOX–CUR–PFOB–PLGA group. The fluorescence intensity was significantly higher in the DOX–CUR–PFOB–PDT group compared with that in the control group (*P* < 0.001), quantitative analysis of the fluorescence intensity of Caspase-3 demonstrated the same conclusion (Fig. 7i), further confirming the excellent effect of intracellular promotion of apoptosis.

### Photochemical-responsive DOX–CUR–PFOB–PLGA NPs possess superior ultrasound imaging

To further investigate the advantages of the photochemical-responsive DOX–CUR–PFOB–PLGA NPs, we checked their in vitro ultrasound imaging effects. Ultrasound imaging is a clinically common non-invasive real-time diagnostic technique that allows for real-time monitoring with low cost and high safety^29^. The NPs underwent liquid–gas phase change after high intensity focused ultrasound (HIFU) irradiation to form microbubbles that could be seen on ultrasound imaging in both B-mode (Fig. 7j) and imaging mode (Fig. 7k) by ultrasonic diagnostic apparatus. In addition, HIFU has the ability to promote drug release to improve the safety and efficacy of treatment^30^. The good ultrasound imaging effects of our NPs may lay a foundation for early tumor diagnosis and treatment.

## Discussion

The cardiotoxicity and side effect of DOX have been a great concern, as cardiovascular disease has been found to be the second ranking cause of morbidity and mortality in cancer patients^31,32^. The pathogenesis of cardiotoxicity related to DOX is sophisticated. DOX can interfere mitochondrial oxidative metabolism by binding to topoisomerase 2β (Top2β) and DNA in cardiomyocytes to yield a ternary complex that can lead to necrotic fibrosis in cardiomyocytes, thereby inducing cardiotoxicity^33^. In recent years, much effort has been devoted to mitigating DOX-related cardiotoxicity. PDT has favorable biocompatibility and has been widely used^34^. Using precise laser source positioning, PDT can selectively aggregate photosensitizers. When combined with DOX, PDT can reduce the toxicity of DOX to normal tissues by enhancing tumor selectivity and increasing cellular uptake of DOX. Most current therapeutic modalities use strategies to selectively enrich DOX in the tumor site to enhance drug uptake by tumor cells. For example, the multiligand-modified DOX micelles prepared by Wang et al.^35^ effectively suppressed the growth of progesterone (PR), estrogen (ER) or human epidermal growth factor receptor 2 (HER2) breast cancers. Furthermore, the hypoxic environment within the tumor microenvironment promotes the rapid growth of tumors and the abnormal neovascular network. Increasing the oxygen levels in the tumor microenvironment could inhibit tumor growth. Moreover, it has been shown^36^ that liposome-mediated phototherapy encapsulated with indocyanine green (ICG) and PFOB has a desirable lethal impact on breast cancer cells in vivo and in vitro. Combining the advantages of the above therapeutic options might achieve even stronger therapeutic effects. Therefore, we fabricated a type of photochemical-responsive NPs, DOX–CUR–PFOB–PLGA, by co-loading chemotherapeutic drug DOX, photosensitizer, and chemosensitizer CUR, and PFOB, which has excellent oxygen affinity onto PLGA which is polymer matrix and has optimal biocompatibility, through a modified double emulsification method. During the preparation process, dehydrochlorination of DOX hydrochloride was achieved through an acid–base neutralization reaction. The results showed that PLGA is an effective drug carrier, as it achieved DOX encapsulation rate up to (70.80 ± 0.47)%. Moreover, the NPs overcame the hydrophobicity of CUR with increased water solubility.

In PDT, a photosensitizer is irradiated with an appropriate wavelength and excited from the ground state to the excited state and thus transfers the energy to oxygen molecules (mainly singlet oxygen), producing ROS. The accumulated ROS has a strong reactive activity boost cellular DOX uptake, resulting in suppressed tumor cell migration and enhanced apoptosis^37^. For instance, when PDT is combined with low-dose cisplatin, it has better killing effects without serious side effects^38^. As another example, when MDA-MB-231 cells were incubated with the photosensitizers, evodiagenine (EVO), and applied to PDT, the cell growth was significantly inhibited and the intracellular ROS level was greatly increased^39^. Consistently, in the current study, we showed that the combination of DOX–CUR–PFOB–PLGA NPs with PDT decreased the viability and skeleton-forming ability of MCF-7 cells significantly. On the one hand, the cytoskeleton is involved in a variety of biological activities, including the maintenance of cell morphology, motility, and cytoplasmic cytoderm synthesis^40^. On the other hand, degradation of the cytoskeleton can restrain the migration of MCF-7 cells. We also showed that the combination of DOX–CUR–PFOB–PLGA NPs with PDT significantly increased the intracellular ROS content, promoted the cellular DOX uptake, and decreased the proliferative capacity of the cells.

DOX can inhibit tumor growth mainly by inducing apoptosis and necrosis of tumor cells through mechanisms such as downregulating BCL-2 protein levels^41^. Hypoxia-inducible factor 1 (HIF-1α is a nuclear transcription factor whose expression in the cells could be induced by the hypoxic microenvironment. A previous study has demonstrated that high expression of HIF-1α is significantly correlated with poor patient prognosis, and HIF-1α could contribute to tumor growth by recruiting tumor-associated macrophages (TAM) and myeloid suppressor cells (MDSC)^42^. The PI3K/AKT signaling pathway is also aberrantly activated in malignant tumors, and the AKT protein, as a signaling hub protein, is phosphorylated and activated by membrane translocation that could catalyze the depolymerization of downstream BCL-2 and BAX to a free state^43^. The activated BCL-2 and BAX can trigger downstream apoptosis-related proteins. Likewise, Caspase-3 serves as a key molecule in the apoptosis process. When Caspase-3 is activated, it plays a pro-apoptotic role^44^. In our study, the ratios of cells in the early and late apoptosis stages were 3.80% and 8.34% in the free DOX group, 3.49% and 14.11% in the DOX–CUR–PFOB–PLGA group, and 21.57% and 20.66% in the DOX–CUR–PFOB–PLGA–PDT group, respectively. In addition, oxygenated nanoparticles co-cultured with MCF-7 cells showed reduced expression of HIF-1α after laser irradiation due to the presence of PFOB with excellent oxygen affinity and the expression of BAX/BCL-2 and cleaved Caspase-3 proteins were upregulated by decreasing the expression of pAKT. Besides, nanoparticles encapsulated with PFOB have been investigated for the development of multiple imaging modalities such as ultrasound, CT and MRI^45^. Our NPs can undergo liquid–gas phase change under the action of HIFU to achieve a good ultrasound imaging effect, bringing new hints for multimodal imaging-guided PDT.

In summary, the DOX–CUR–PFOB–PLGA NPs with laser irradiation at an intensity of 40 mW/cm^2^ for 150 s exhibited significantly higher cytotoxicity on MCF-7 cells compared to the free DOX group and the DOX–CUR–PFOB–PLGA group without laser irradiation. There are some limitations of this study. First, the influence of the photochemical-responsive NPs was only tested on MCF-7 cells. Second, the development of multi-drug resistance (MDR) is an obstacle for effective treatments. While in this study, we showed that the NPs may have the same imaging effect comparable to CT and MRI, and PDT could reverse tumor MDR; however, the underlying mechanisms remain unclear. Besides, in recent years, there has been growing interest in developing multimodal imaging-guided combination therapy with sensitive chemotherapeutic agents. We will continue to pursue aspects pertaining to multimodal imaging-guided NP-mediated PDT that can reverse drug resistance in breast cancer in future studies.

## Materials and methods

### Main reagents

CUR (≥94%), PFOB, polyvinyl alcohol (PVA), DAPI staining solution, TritonX-100 were purchased from Sigma-Aldrich (MO, USA); PLGA (50:50, molecular weight [MW] = 12,000) was purchased from Daigang Biomaterials Co., Ltd(Jinan, China); DOX hydrochloride (97%) was purchased from Aladdin; dichloromethane (DCM), isopropanol and triethylamine were purchased from Chemical Book (Beijing, China); DMEM medium and fetal bovine serum (FBS) were purchased from Gibco (MA, USA); Live/Dead cell kit and Alexa Fluor 594 phalloidin were purchased from Thermo Fisher; BCA protein concentration assay kit and ROS assay kit were purchased from Beyotime; CCK-8 kit and antibodies for AKT, pAKT, cleaved Caspase-3, BAX, BCL-2, HIF-1α and β-actin were purchased from Abcam (Cambridge, UK).

### Synthesis of CUR–PFOB–PLGA and DOX–CUR–PFOB–PLGA NPs

Briefly, CUR (2 mg), PLGA (5 mg), PFOB (100 μL), and DCM (2 mL) were mixed and dissolved completely in an ultrasonic cleaner. The mixture was then emulsified for 1 min using an ultrasonic probe at a power density of 100 W. Then, the mixture was sonicated for a second time for 1.5 min by adding 5 mL of PVA (4%). Then, 10 mL of isopropanol (2%) was added and magnetically stirred for 3 h to evaporate the organic solvent. The mixture was purified by centrifugation (1000 rpm, 5 min) twice to achieve the CUR–PFOB–PLGA NPs and lyophilized to powder for future use. Since DOX hydrochloride has good aqueous solubility, we optimized the DOX–CUR–PFOB–PLGA NPs further. Briefly, a mixture of 3 mL of DCM containing DOX hydrochloride (10 mg) and triethylamine (at a molar ratio of 1:2) and PLGA (100 mg) and a mixture of 2 mL of DCM containing CUR (10 mg) and PFOB (100 μL) was slowly and dropwisely added to 20 mL of PVA (1%) and re-emulsified at a power intensity of 250 W. The rest of the procedures was the same as that of the CUR–PFOB–PLGA NPs. All the ultrasonic emulsification processes were performed under ice bath conditions.

### Characterization of nanoparticles

The morphology of the composite NPs was observed using TEM and SEM. The lyophilized NPs were diluted in xxx at a certain concentration and 10 drops (each drop ~ 10 μL) were loaded on coverslips and observed under an inverted fluorescence microscope. The size, dispersion and Zeta potential of the NPs were analyzed by DLS. The chemical composition was validated by FTIR. Also, the NPs dissolved in ultrapure water were perfused with nitrogen to fully dislodge oxygen, and then infused with oxygen for 10 min. Changes in oxygen concentration were measured using a portable dissolved oxygen meter (ultrapure water group was used as the control.). Moreover, the absorbance peaks of DOX and CUR in the supernatant were measured using the UV spectrophotometer and HPLC to calculate the EE and LE, respectively, where EE = (total drug-free drugs)/total drugs × 100% and LE = (total drugs-free drugs)/total nanoparticles × 100% (all in mass units).

### Release of nanoparticles in vitro

Certain doses of the CUR–PFOB–PLGA and DOX–CUR–PFOB–PLGA NPs were dissolved in 5 mL PBS in a dialysis bag and the dialysis bags were placed in PBS solution (40 mL, pH = 7.4) prior to and post photochemical activation, respectively. After the NPs were dispersed at 100 rpm at 37 °C, 1 mL of liquid from the release medium was taken at a predetermined time point, and 1 mL of fresh PBS was replenished. The cumulative release rate of DOX and CUR was measured using UV and HPLC. The DOX and CUR release curves were then fitted using the Origin 2021 software.

### Cell culture

MCF-7 human breast cancer cells were purchased from the American Type Culture Collection (ATCC, Manassas, VA, USA), cultured in Dulbecco's modified Eagle medium (DMEM) containing 10% (v/v) FBS, 100 IU/mL penicillin, 10 mM MEM non-essential amino acids (NEAA) and 100 mM sodium pyruvate and humidified at 5% CO_2_ and 37 °C.

### Biocompatibility of nanoparticles

The cytotoxicity of different treatments, including CUR, DOX, CUR–PFOB–PLGA and DOX–CUR–PFOB–PLGA with or without different intensities of laser irradiation, was assayed using the Cell Proliferation Toxicity Assay (CCK-8) and the OD value at 450 nm was measured on a multifunctional analyzer. In addition, a live/dead cell viability/cytotoxicity assay kit and skeleton staining method were employed to further characterize the impact of NPs on cell activity. Briefly, MCF-7 cells in different groups, including the control group, free DOX group, CUR–PFOB–PLGA group, DOX–CUR–PFOB–PLGA group, CUR–PFOB–PLGA–PDT (CUR–PFOB–PLGA NPs with laser irradiation) group, and DOX–CUR–PFOB–PLGA–PDT group were cultured in different dishes. Twenty-four hours later, dishes containing cells of the control group and laser irradiation group were replaced with fresh medium and the rest dishes were incubated with different drugs for 4 h, followed by laser irradiation for 150 s. In the next 24 h, 200 L of hybrid dye was added and the cells were stained for 20 min. At the corresponding time points, cells were washed with PBS, fixed in 4% paraformaldehyde and permeabilized in PBS containing 0.1% (v/v) Triton X-100. The actin and nuclei were stained with 200 μL Alexa Fluor 594 phalloidin (5 μg/ml) and 4,6-diamidino-2-benzoindolyl dilaurate (DAPI, 10 μg/ml), respectively, and then observed under LSCM. All experiments performed in triplicate.

### Intracellular drug uptake

MCF-7 cells at logarithmic growth stage were used for experiments. The cells were cultured in different dishes for different groups: free DOX group, DOX–CUR–PFOB–PLGA group, and DOX–CUR–PFOB–PLGA–PDT group. After being given different treatments, the cells were digested and collected for FCM to measure the relative DOX fluorescence intensity. The experiment was repeated three times and analyzed by Prism.

### Wound healing assay and transwell assay

Cells incubated with different drugs were vertically scratched along the bottom horizontal line with a sterile pipette tip (200 μL) before laser irradiation. The initial scratched areas were recorded under the light microscope after PBS wash. After laser irradiation for 150 s, the scratched areas were recorded again at 24 h and 48 h. Briefly, cells were starved with 0.5% serum medium for 12 h and 5 × 10^4^ cells per well were inoculated in chambers while 800 μL of complete medium was added to the bottom of the wells. After 24 h, the inserts were fixed with 4% paraformaldehyde, stained with 0.1% crystal violet at room temperature, washed with ultrapurewater, gently wiped off with a cotton swab and placed in a new 24-well plate to monitor cell migration. The above were photographed under the inverted fluorescence microscope and analyzed by Image J.

### Colony formation assay

To investigate how the proliferation ability of cells was affected in the control, free DOX, DOX–CUR–PFOB–PLGA and DOX–CUR–PFOB–PLGA–PDT groups. The cells were treated and digested 24 h later. The cells were inoculated again into 3.5 mm dishes (800 cells/dish), which were cultured for 14 d and then stained with 0.1% crystalline violet for 3 min. Experimental groupings were the same in the following. Eventually the basal plane was photographed with a digital camera and the colony counts were calculated by Image J.

### Generation of ROS

Cells were kept in the dark for 30 min after different treatments. The cells were washed with PBS and incubated with DCFH-DA (10 μmol/L) for 20 min in the dark. Then, the cells were washed again using the medium without fetal bovine serum. Finally, the cells were observed under the inverted fluorescence microscope. Additionally, the intracellular DCFH fluorescence intensity was also detected by FCM and analyzed by Image J.

### Apoptosis assay

Cells were washed with pre-cooled PBS and then incubated with 10 μL of Annexin V-FITC and 5 μL PI for 10 min. The apoptosis rate was assessed with FCM.

### Western blot

Total proteins extracted from RIPA were quantified with the BCA protein assay kit. Next, sample proteins were subjected to SDS electrophoresis followed by transferring onto a PVDF membrane (0.45 μm). 5% BSA was used to block unspecific binding for 1 h. Cropped the membrane into suitable fragments according to the size of the molecular and the membrane were incubated overnight at 4 °C with the relevant primary antibodies including AKT(1:1000), pAKT (1:1000), β-actin (1:1000), BAX (1:1000), BCL-2 (1:1000), cleaved Caspase-3 (1:500) and HIF-1α (1:200). Then, TBS washed and an HRP-conjugated secondary antibody (1:10,000) was applied to the membrane, and detected by an enhanced chemiluminescence system. The target proteins were normalized by β-actin and the antigen–antibody complexes were further quantified by Image J.

### Immunofluorescence staining

After 24 h, cells in different groups were fixed with 4% paraformaldehyde for 20 min and then permeabilized with 0.1% Triton-100 for 15 min. After washing with PBS, the cells were incubated with Caspase-3 antibody (1:250) overnight at 4 °C. After washing with PBS, the cells were incubated with a fluorescent-labeled secondary antibody (1:1000) at 37 °C for 1 h. After washing with PBS, the nuclei were stained with DAPI. Then, the localization of Caspase-3 was analyzed under LSCM. All studies were carried in triplicate.

### Ultrasound imaging in vitro

Using agarose gels as raw material to manufacture perforated gel models in which DOX–CUR–PFOB–PLGA solution (1 mg/mL) can be placed and irradiated with HIFU (120 W, 5 s). The ultrasound imaging images in the B-mode and imaging mode were obtained by ultrasonic diagnostic apparatus.

### Statistical analysis

In this study, each experimental section was repeated three times, and the result of each study was expressed as mean ± standard deviation (SD). Data were performed applying the Student’s t-test and one-way ANOVA analysis to examine the differences among variables. All statistical quantifications were performed with Image J 1.46 r, GraphPad Prism 8.0 and Origin 2021 Software.

### Ethics statement

The studies not involving human participants and animals were not required to be reviewed and approved by the ethics committee.

## Supplementary Information

Supplementary Information.

## Data Availability

The datasets used and/or analyzed during the current study are available upon reasonable request.
