# Author Correction: A photochemical-responsive nanoparticle boosts doxorubicin uptake to suppress breast cancer cell proliferation by apoptosis

**DOI:** 10.1038/s41598-023-28014-3

**Published:** 2023-01-19

**Authors:** Ying Zhang, Kaiting Li, Xiaoyu Han, Qing Chen, Lan Shao, Dingqun Bai

**Affiliations:** 1 Department of Rehabilitation Medicine, The First Afliated Hospital of Chongqing Medical University, Chongqing, 400016 China; 2 The Chongqing Key Laboratory of Translational Medicine in Major Metabolic Diseases, The First Afliated Hospital of Chongqing Medical University, Chongqing, China

Correction to: *Scientific Reports* 10.1038/s41598-022-14518-x, published online 20 June 2022

The original version of this Article contained an error in the Funding section.

“This work was supported by National Natural Science Foundation of China (81871583, 82003306), Natural Science Foundation of Chongqing (cstc2019jcyj-msxmX0397, cstc2020jcyj-msxmX0484) and Cultivating Fund in the First Affiliated Hospital of Chongqing Medical University (PYJJ2018-24, PYJJ2019-214).”

now reads:

“This work was supported by National Natural Science Foundation of China (81871853, 82003306), Natural Science Foundation of Chongqing (cstc2019jcyj-msxmX0397, cstc2020jcyj-msxmX0484) and Cultivating Fund in the First Affiliated Hospital of Chongqing Medical University (PYJJ2018-24, PYJJ2019-214).”

The original Article has been corrected.

