# Author Correction: A photochemical-responsive nanoparticle boosts doxorubicin uptake to suppress breast cancer cell proliferation by apoptosis

**DOI:** 10.1038/s41598-023-41335-7

**Published:** 2023-09-04

**Authors:** Ying Zhang, Kaiting Li, Xiaoyu Han, Qing Chen, Lan Shao, Dingqun Bai

**Affiliations:** 1 Department of Rehabilitation Medicine, The First Affiliated Hospital of Chongqing Medical University, Chongqing, 400016 China; 2 The Chongqing Key Laboratory of Translational Medicine in Major Metabolic Diseases, The First Affiliated Hospital of Chongqing Medical University, Chongqing, China

Correction to: *Scientific Reports* 10.1038/s41598-022-14518-x, published online 20 June 2022

The original version of this Article contained errors in Fig. 7, where sample number images in panels (**j**) and (**k**) were replaced incorrectly during figure assembly. The original Fig. 7 and accompanying legend appears below.

**Figure 7:**
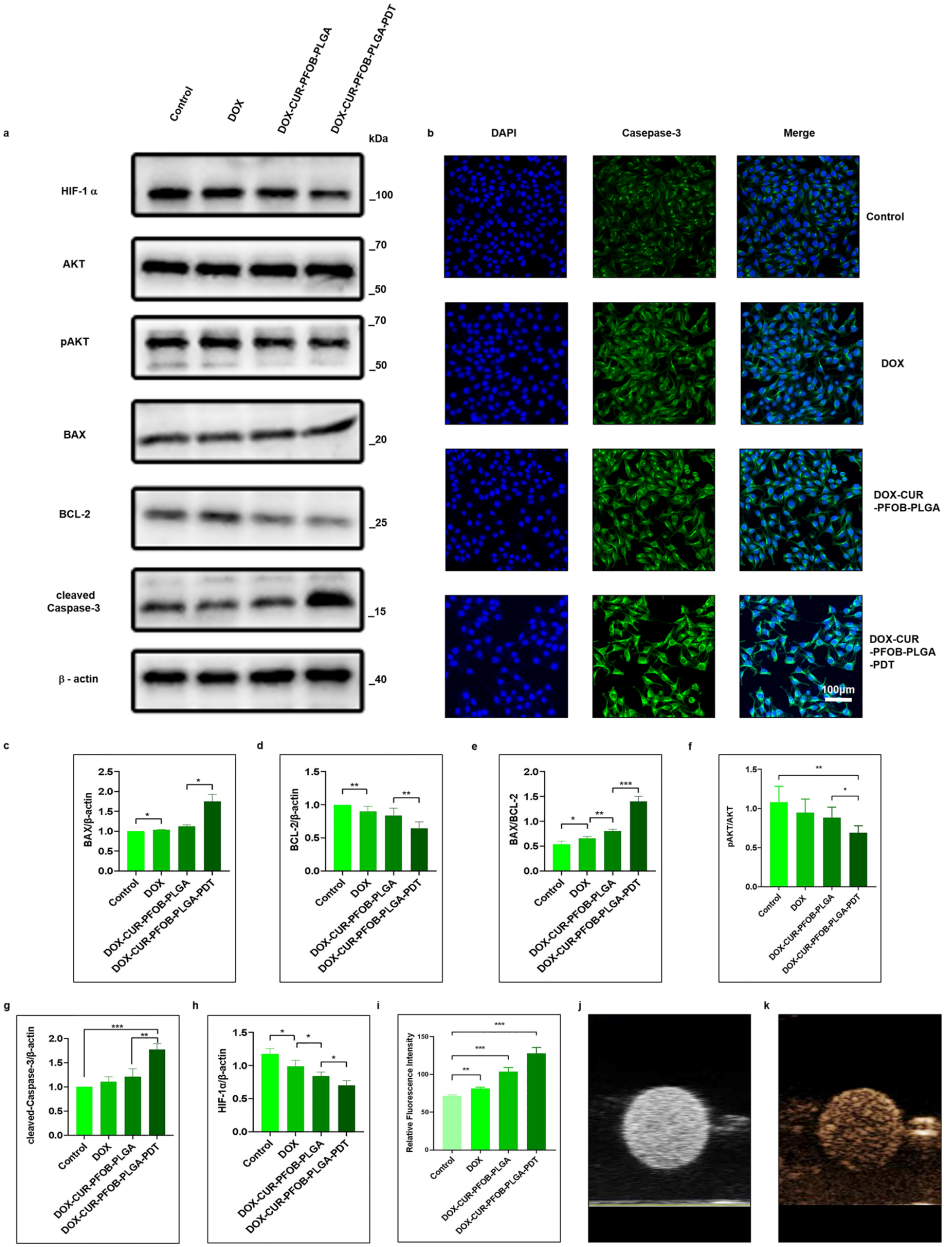
Molecular biology detection of MCF-7 cells and ultrasound imaging of DOX–CUR–PFOB–PLGA NPs by HIFU. (**a**) Western blot detection for HIF-1α, AKT, p-AKT, BAX, BCL-2, cleaved Caspase-3 and β-actin protein expressions, The full-length gels of all cropped blots combined are shown in Supplementary Figure S1; (**b**) The effects of DOX–CUR–PFOB–PLGA NPs on the expression of Caspase-3 (green) in MCF-7 cells incorporated with DOX, DOX–CUR–PFOB–PLGA or DOX–CUR–PFOB–PLGA and PDT, The nucleus was counterstained with DAPI (blue). (c) Quantitative analysis of the expression of BAX/β-actin; (**d**) Quantitative analysis of the expression of BCL-2/β-actin; (e) Quantitative analysis of the expression of BAX/BCL-2; (**f**) Quantitative analysis of the expression of pAKT/AKT; (**g**) Quantitative analysis of the expression of cleaved Caspase-3/β-actin; (**h**) Quantitative analysis of the expression of HIF-1α/β-actin. (**i**) Quantitative analysis of the relative fluorescence intensity of Caspase-3; (**j**) The B-mode image of DOX–CUR–PFOB–PLGA NPs by ultrasonic diagnostic apparatus; (**k**) The imaging mode image of DOX–CUR–PFOB–PLGA NPs by ultrasonic diagnostic apparatus. The expression of the proteins was quantified by Image J 1.46 r (https://imagej.nih.gov/ij/docs/guide/146.html) and Graphpad Prism 8.0 (https://www.graphpad.com/updates), *P < 0.05; **P < 0.01; ***P < 0.001.

The original Article has been corrected.

