# Supplementary material for: A photochemical-responsive nanoparticle boosts doxorubicin uptake to suppress breast cancer cell proliferation by apoptosis
Source: Sci Rep. 2022 Jun 20;12:10354. doi: 10.1038/s41598-022-14518-x (PMC9209492; doi:10.1038/s41598-022-14518-x)
Supplement: Supplementary file 1 — Supplementary Information. [file 41598_2022_14518_MOESM1_ESM.pdf]

A photochemical-responsive nanoparticle boosts doxorubicin uptake to suppress breast cancer cell proliferation by apoptosis

Ying Zhang[1], Kaiting Li[1], Xiaoyu Han[1], Qing Chen[1], Lan Shao[1], Dingqun Bai[1,*]

[1] Department of Rehabilitation Medicine, The First Affiliated Hospital of Chongqing Medical University, Chongqing 400016, P.R. China

[*] Corresponding author: Dingqun Bai, Department of Rehabilitation Medicine, The First Affiliated Hospital of Chongqing Medical University, Chongqing 400016, P.R. China.

Tel: +86 13808380876

Email: baidingqun2014@163.com

Ying Zhang: 2640458788@qq.com

Kaiting Li: likaiting1949@163.com

Xiaoyu Han:419765622@qq.com

Qing Chen:cqcq20123634@163.com

Lan Shao: shaolanfyy@163.com

Supplementary Figure S1

The merged full gels for western blot images used to create Figure 7a are shown. Four experimental groups (Control、DOX、DOX-CUR-PFOB-PLGA and DOX-CUR-PFOB-PLGA-PDT) were performed on one gel three times, and markers were added to the first, sixth and eleventh lanes to facilitate cropping against the corresponding molecular weights prior to hybridization with antibodies including AKT (50- below 70 kDa)、HIF-1 α (above 70 kDa)、pAKT (50-70 kDa)、β-actin (35 or 40- below 50 kDa)、BAX (10-below 35 kDa)、cleaved Caspase-3 (10-below 35 kDa) and BCL-2 (10-below 35 kDa). Black dotted lines mark the crop marks (i.e. the dividing line between different gels) and red rectangles mark the bands used.

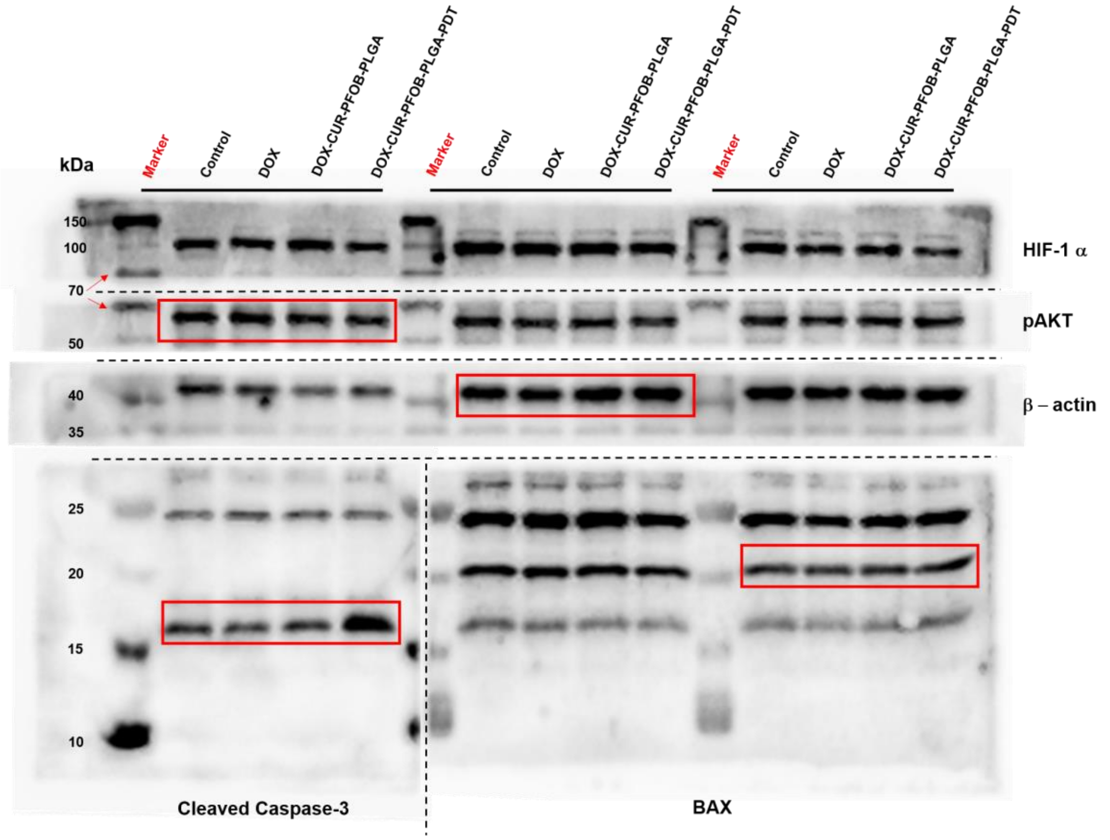

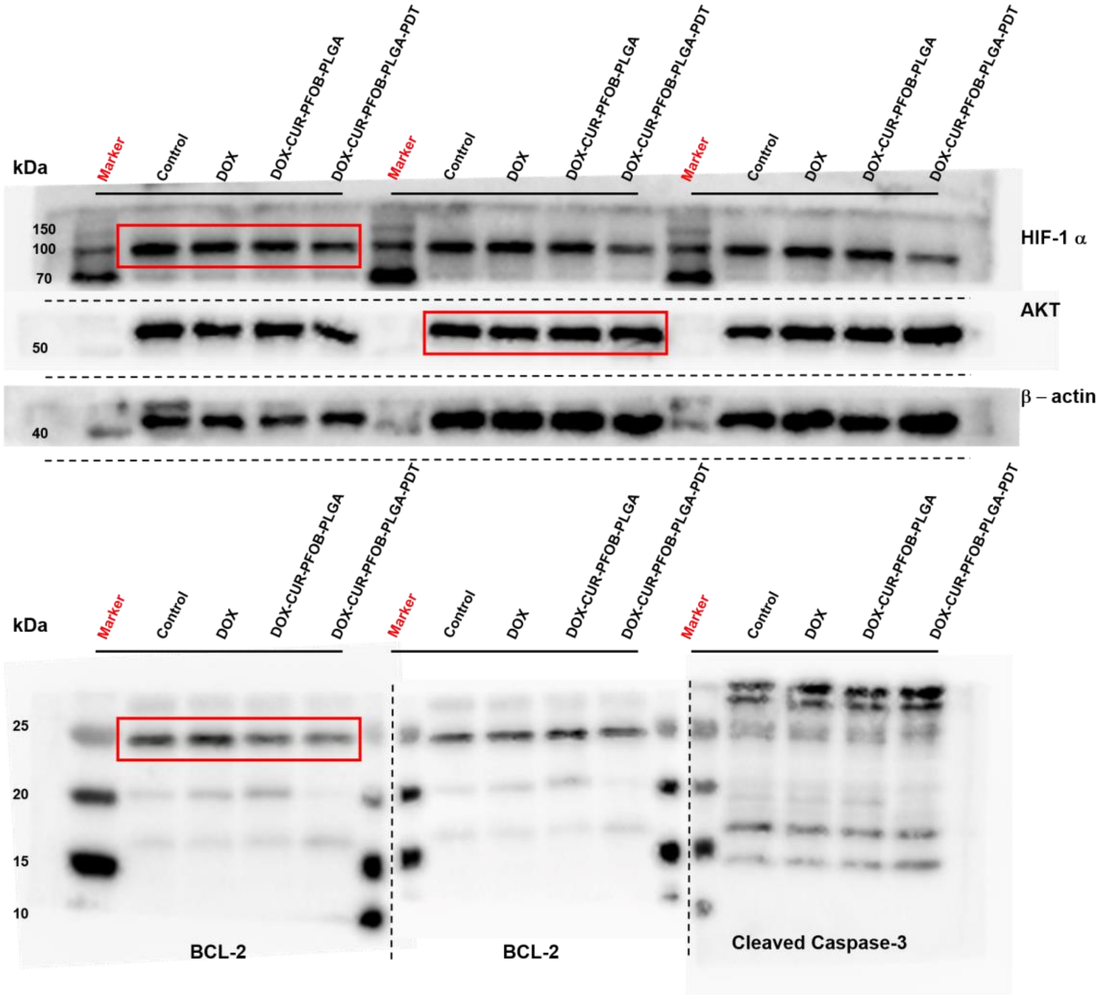
kDa
Marker
Control
DOX
DOX-CUR-PFOB-PLGA
DOX-CUR-PFOB-PLGA-PDT
150
100
70
HIF-1 α
AKT
50
β – actin
40
25
20
15
10
BCL-2
BCL-2
Cleaved Caspase-3
